# Cervical Ectopic Pregnancy: Combination Treatment With Multi-Dose Methotrexate Regimen, Uterine Artery Embolization, and Suction Curettage

**DOI:** 10.7759/cureus.52125

**Published:** 2024-01-11

**Authors:** Larissa L Aroche Gutierrez, Jason Bunn, Gabriele Duvernois, Courtney Baker

**Affiliations:** 1 Obstetrics and Gynecology, University of Texas Southwestern Medical Center, Dallas, USA

**Keywords:** fertility-sparing treatment, suction curettage, uterine artery embolization, multi-dose methotrexate, cervical ectopic

## Abstract

Cervical ectopic pregnancy is a rare condition associated with significant morbidity. With early ultrasound, fertility preservation options have become more common. No consensus on treatment exists, but many treatment modalities have been reported with good outcomes. This case report exemplifies the advantages of combination treatment for a patient with difficult outpatient follow-up, achieving a rapid resolution without increased morbidity.

## Introduction

Ectopic pregnancies account for one to two percent of all pregnancies but are the main cause of direct obstetric deaths in the first trimester [1]. Non-tubal ectopic pregnancies can be challenging to diagnose and are subsequently associated with more complications. While fewer than one percent of ectopic pregnancies are cervical [2], morbidity associated with cervical ectopic pregnancy is high; the rate of hysterectomy has been reported to be nine percent in a review of 454 cases [3]. While there is no consensus on the treatment of cervical ectopic pregnancies, systemic or local methotrexate is commonly used alone or in combination with other treatments. Uterine artery embolization (UAE) with methotrexate has been reported with good clinical outcomes in case series [4-5]. UAE followed by curettage has also been shown to be effective in a retrospective study [6]. This case demonstrates a methodical approach to using all three treatment modalities based on clinical factors, such as difficult outpatient follow-up. Furthermore, the total treatment length was short compared to those in other reports, and good patient satisfaction was obtained, with no increased morbidity to the patient.

## Case presentation

**Figure 1:**
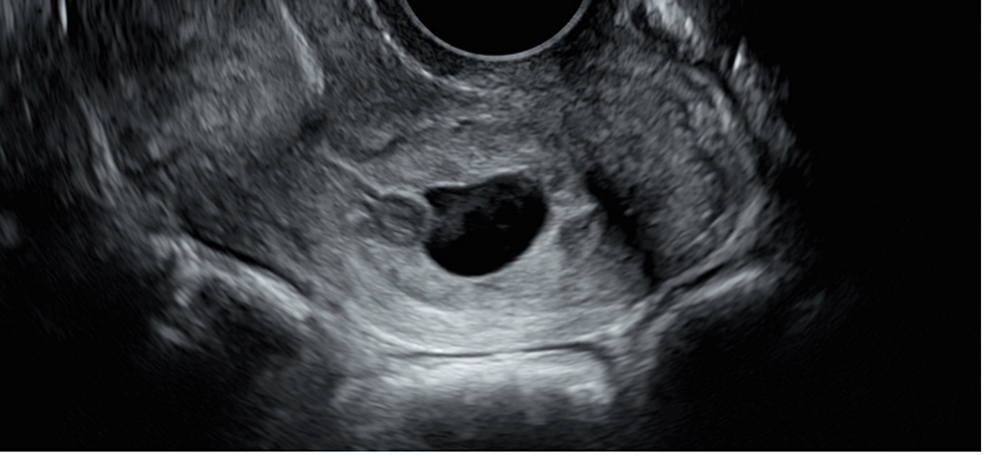
Transvaginal pelvic ultrasound at initial presentation showing a cervical ectopic pregnancy.

The patient is a 24-year-old, gravida-2 para-0 abortus-1, Hispanic female with no significant medical history, social, family, or prior gynecologic surgical history who presented to the emergency department at four weeks four days of gestation by the last menstrual period (LMP). She reported vaginal bleeding for one day with passage of clots and tissue, which then decreased to vaginal spotting with associated mild lower abdominal cramping that self-resolved. Physical exam revealed a non-tender abdomen, no active vaginal bleeding, non-dilated cervix, and cervical motion tenderness. Her hemoglobin (Hgb) was 13.9 g/dL and initial serum beta human chorionic gonadotropin (B-hCG) value was 36,461 mIU/mL. Pelvic ultrasound imaging showed a 1.5 cm gestational sac and yolk sac with surrounding blood flow located in the cervical canal 20 mm from the external cervical os (Figure 1). Differential diagnosis included ongoing expulsion of a failed intrauterine pregnancy although cervical ectopic pregnancy could not be excluded. Given the resolution of symptoms, she was discharged home with close follow-up and strict return precautions.

The patient returned for follow-up in the office setting five days later and denied any further symptoms. A repeat B-hCG level was 48,341 mIU/mL. The patient did not attend a planned one-week follow-up for repeat pelvic ultrasound and labs, and she was not able to be contacted despite multiple attempts. 

The patient re-presented to the emergency department at eight weeks six days of gestation by LMP with increased vaginal bleeding with clots and intermittent lower abdominal cramping for two days. Her abdomen was non-tender. There was 5 mL of pooled blood in the vagina on speculum exam. The B-hCG level was 9,875 mIU/mL, and Hgb was unchanged at 13.4 g/dL. 

Thrombocytopenia was noted (platelets 59 x 109/L). Hematology consultation determined her low platelets to be pseudo-thrombocytopenia due to extensive platelet clumping seen on the peripheral blood smear. Since the platelet count in the citrate tube was >100 x 109/L, this further supported the diagnosis of pseudothrombocytopenia, and the hematology consulting team did not recommend further follow-up. It was determined, with assistance of the hematology consultants, that methotrexate could be administered in the absence of absolute contraindications. Notably, the patient’s platelet count on the day of discharge were within the normal limits.

**Figure 2:**
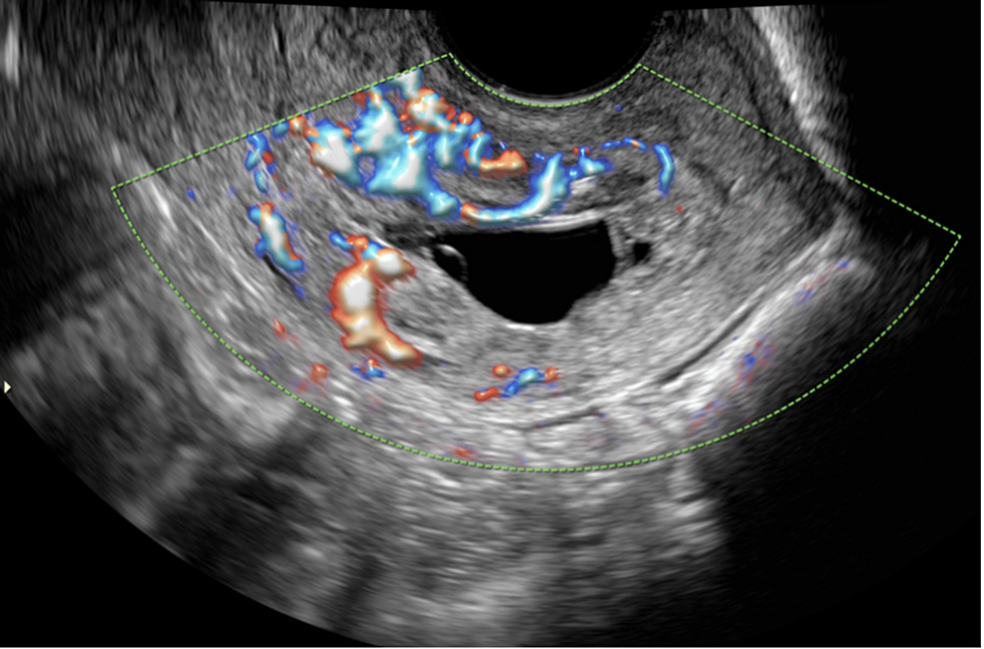
Transvaginal pelvic ultrasound at representation with trophoblastic tissue at the cervix and marked vascularity.

Repeat pelvic ultrasound showed an irregularly shaped gestational sac with a yolk sac in the cervical canal 9 mm from the external os, and increased vascularity confirmed trophoblastic implantation in the cervix consistent with cervical ectopic pregnancy (Figure 2). After a thorough medical counseling, the patient was admitted to receive multi-dose methotrexate (MTX) 1 mg/kg/m^2^ on days one, three, five, and seven with leucovorin rescue 0.1 mg/kg on days two, four, six, and eight. The B-hCG level declined to 7,031 mIU/mL on day two of admission. Given the hypervascularity of the gestational sac on pelvic ultrasound and thrombocytopenia, there was concern for future hemorrhage. Upon discussion with the patient and consultation with Interventional Radiology, including counseling about pregnancy risks after UAE, the decision was made to proceed with UAE to reduce risk of recurrent bleeding. An uncomplicated UAE with Gelfoam with 10 mL estimated blood loss was performed on hospital day four. The patient received 24 hours of IV piperacillin/tazobactam post-operatively for prophylaxis. The B-hCG level declined to 5,150 mIU/mL on hospital day five after the procedure.

**Figure 3:**
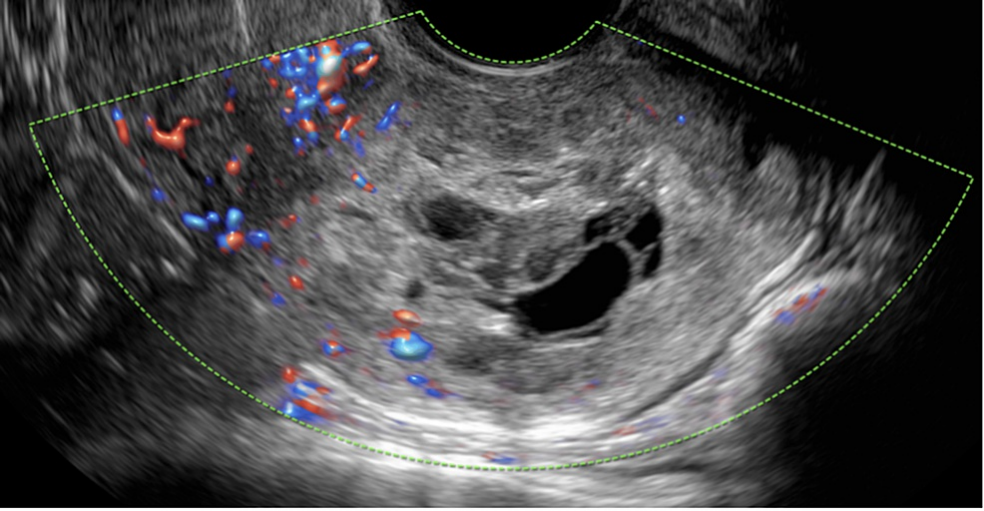
Transvaginal pelvic ultrasound following multi-dose methotrexate regimen and uterine artery embolization.

The patient continued to have dark-colored vaginal spotting and intermittent passage of small blood clots. Lab results were trended while she received the multi-dose MTX/leucovorin regimen; Hgb decreased to 10.6 g/dL, and there was a mild increase in alanine transaminase (ALT) and aspartate transaminase (AST) (peak values 76 and 55 U/L, respectively). A pelvic ultrasound was repeated following four doses of MTX with leucovorin rescue on hospital day eight at which time the B-hCG was 2,207 mIU/mL. The ultrasound demonstrated a persistent irregular and hemorrhagic gestational sac measuring 2.7 cm within the cervical canal with surrounding decreased vascularity compared to imaging eight days prior (Figure 3). The yolk sac was no longer visualized.

Although decreasing B-hCG levels indicated ongoing resolution from prior treatment with MTX and UAE, there was concern by the patient and medical team about the persistent gestational sac. Imaging and lab results, clinical stability, prior outpatient compliance, and patient comfort were weighed through shared decision-making with the patient. The decision was made to proceed with a suction curettage to remove the gestational sac and expedite resolution in an inpatient setting. An uncomplicated ultrasound-guided suction curettage with 20 mL estimated blood loss was performed with spinal anesthesia on hospital day nine. After aspiration, a 16-French foley bulb filled with 25 cc saline was inserted prophylactically into the endocervical canal for hemostasis. Serial exams and labs revealed no ongoing bleeding overnight. No further vaginal bleeding occurred following removal of the foley bulb on post-operative day one. 

Surgical pathology showed products of conception from the suction curettage. The B-hCG continued to downtrend to 237 mIU/mL on hospital day 10. The patient was counseled about the importance of pregnancy prevention until resolution of her cervical ectopic pregnancy was confirmed; she opted to receive depot-medroxyprogesterone acetate for contraception. She was comfortable with discharge and confirmed the importance of close follow-up until resolution of this episode. She continued follow-up outpatient on a weekly basis until her B-hCG was negative three weeks later.

## Discussion

Existing evidence on the treatment of cervical ectopic pregnancy is limited but helped guide the management in this case. First, given a peak B-hCG of nearly 50,000 mIU/mL, MTX alone may have failed to resolve the pregnancy. Local MTX is more likely to fail in cases when B-hCG equals or exceeds 10,000 mIU/mL based on prognostic factors published in a retrospective study [7]. Retrospective analyses have also reported a high likelihood of systemic single-dose methotrexate failure when B-hCG is above 7,000 mIU/mL [8]. In addition, a lengthy time to serum hCG resolution has been reported with this single treatment modality; a retrospective study of five cases treated with single high-dose MTX reported a median time of 68 days [9].

Regarding UAE, treatment with resorbable media for cervical ectopic pregnancy has had good fertility outcomes according to two previous studies [4-5]. While blood flow around the gestational sac can be used to differentiate a cervical ectopic pregnancy from a low-lying pregnancy or inevitable abortion [5,8,10], no clinical guidance exists regarding the use of UAE based on the degree of vascularity surrounding a cervical ectopic pregnancy [4-5]. The hypervascularity surrounding the gestational sac on ultrasound in this case led the team to choose UAE over primary suction curettage. Potential catastrophic hemorrhage may have occurred with the latter, which would have likely resulted in definitive surgical management with a hysterectomy, an undesired outcome for this patient.

Treatment utilizing a combination of MTX, unplanned or planned UAE, and curettage has been reported. A case series of 16 cervical pregnancies included two patients with B-hCG of 45,830 and 25,600 who were treated with MTX, followed by emergency UAE and curettage due to acute bleeding [11]. Two different patients, who had B-hCG of 1,392 and 1,536 and were also treated with MTX, had persistent bleeding and underwent non-emergent UAE and curettage [11]. All four cases resolved in 37 days on average; however, the length of duration was counted from UAE and curettage to undetectable hCG and did not account for the start of the MTX treatment [11]. A retrospective study similarly showed a mean hospital stay of 6.8 days in 19 patients who underwent UAE and curettage; three of these patients received intramuscular, parametrial, or intra-arterial MTX injections [12]. The study did not report time to undetectable B-hCG. A case series of 13 patients who received transcatheter intra-arterial MTX infusion combined with UAE followed by dilation and curettage reported a median of 35.9 +/ 6.2 days to undetectable B-hCG [13].

Other treatment modalities have been reported. In a retrospective review, suction curettage with balloon tamponade was the single regimen used for resolution of 13 cervical ectopic pregnancy cases; however, this technique is largely limited by surgeon expertise [14]. In addition, the minority of the cases had B-hCG >9,000 mIU/mL, and most patients had either no symptoms or minimal vaginal spotting. One recently published case report of a cervical ectopic pregnancy with an embryonic pole utilized three modalities, namely, intracardiac KCl injection, cervical ripening balloon, and methotrexate, achieving a resolution of the pregnancy after 12 weeks [15].

The case presented was performed in a non-emergent stepwise manner. The total hospital stay was 10 days, and the length to resolution of the episode from start of the multi-dose MTX treatment to negative B-hCG was 38 days. It is unknown what the outcome may have been if the clinical team had proceeded with UAE and suction curettage, without initial MTX therapy. Suction curettage was performed to expedite resolution in an inpatient setting given the patient’s clinical stability, her concern about potential recurrence of vaginal bleeding, and her history of difficulty with outpatient follow-up.

## Conclusions

This case provides unique evidence for a non-emergent and stepwise multimodal treatment resulting in a relatively short duration to undetectable B-hCG levels. The use of multiple modalities and close monitoring may be resource-prohibitive for some populations but, when available, may be preferred by some patients due to decreased time to resolution without increased morbidity. Cervical ectopic pregnancy was successfully resolved, and fertility likely preserved, in this patient using multimodal treatment with uterine artery embolization, multi-dose methotrexate regimen, and suction curettage.
